# From the Editor’s Desk

**Published:** 2015

**Authors:** Pat Commerford

**Affiliations:** Editor-in-Chief

It is difficult for those of us who trained in medicine prior to the advent of the epidemic of HIV/AIDS to explain to junior colleagues just how the epidemic has affected every aspect of medical practice, and the degree of complexity it has added. The timely review by Pillay and colleagues (page 70) of HIV-associated large-vessel vasculopathy, which surveys both the current and emerging spectrum of the condition, as seen in vascular surgical practice, serves to clarify some of the uncertainties around this complex problem. It is difficult to comprehend that the diverse disease spectrum of aneurysms, occlusive disease, spontaneous arteriovenous fistulae and dissections have a unifying pathogenesis and it may well be that further advances in knowledge will lead to re-classification and changes in terminology. The authors, importantly, highlight areas of therapeutic uncertainty, which hopefully will change with advances in the understanding of pathophysiology and a more structured approach to interventions.

Further exploring the relationship of HIV/AIDS with cardiovascular disease, Longo-Mbenza and co-workers (page 52), in a cross-sectional study, examined the relationship between *H pylori* infection and the metabolic syndrome among HIV-infected black Africans. The results, showing that *H pylori* infection was associated with the metabolic syndrome in HIV-infected patients, are intriguing but require confirmation in larger studies involving control subjects from a similar population who are not HIV infected.

The proceedings of the recent meeting of the PASCAR Hypertension Task Force, reported in this issue (page 82), discusses the importance of hypertension in Africa, the reasons for its increase and the urgent need for strategies to limit and control the impact on life-expectancy and health of the population. Notably, the document expresses the importance of involving all relevant groups, societies and organisations with influence in this area.

The publication of the proceedings in this issue provides an opportunity for any individuals or groups who believe that they should have an input into the process, and who have not been involved, to contact PASCAR and voice their interest in participation. Particularly fascinating in the PASCAR report is the declared interest in re-examining the issues of prevention and treatment in the African context rather than simply adopting solutions developed elsewhere.

The proceedings review all the available evidence and express an interest in examining critically relevant issues regarding applicability of results of clinical trials and selection of drug therapy in an African context. This roadmap, if brought to a successful conclusion in the spirit expressed by those initiating it, should have a significant impact on the problem of hypertension in Africa.

It is helpful to be able to publish in the same issue, the article from Magalhaes and colleagues (page 57), which examined the 24-hour urinary sodium excretion and knowledge, attitudes and behaviour regarding salt intake of 123 Angolan medical students at a single medical school in that country. The authors concluded that the level of salt intake was excessive and the behaviour of medical students was inappropriate and inadequate regarding salt intake. The authors are to be congratulated for investigating a difficult issue and for publishing their results. If we do not recognise the deficiencies of our medical educational facilities we will not improve them.

The research and publication by the authors of the Magalhaes article challenge all of us involved in medical student education to re-examine our own students and our own practice. Do we as teachers know how many of our own students smoke, drink excessively or use recreational drugs? The Magelhaes article should be a resounding call to all of us to focus on our trainees, as the attitudes and habits they learn while students will significantly influence their management of patients in the future.

Returning to the opening theme of what I have learned during a career that leaves me astounded at the amount of new information I have had to absorb (and have not done so very well), I report my response to the Mouton article (page 63). Forty years ago, hypertrophic cardiomyopathy was a mystery. We agonised over the physical signs, argued about the haemodynamic characteristics and had little information about the treatment and natural history. The situation is now dramatically different with definitive natural history studies guiding therapeutic choices, and echocardiographic studies establishing unequivocally the phenotypic characteristics and correlating them with genotypic features.

